# Prevention of falls and fractures in old people by administration of calcium and vitamin d. randomized clinical trial

**DOI:** 10.1186/1471-2458-11-910

**Published:** 2011-12-09

**Authors:** Jesús López-Torres Hidalgo

**Affiliations:** 1 Unidad de Investigación de la Gerencia de Atención Primaria de Albacete (Servicio de Salud de Castilla-La Mancha), Marqués de Villores 6-8, 02001 Albacete, Spain

## Abstract

**Background:**

There are many studies that associate vitamin D serum levels in older persons with muscle strength, physical performance and risk of fractures and falls. However, current evidence is insufficient to make a general recommendation for administrating calcium and vitamin D to older persons. The objective of this study is to determine the effectiveness of calcium and vitamin D supplementation in improving musculoskeletal function and decreasing the number of falls in person aged over 65 years.

**Methods/Design:**

Phase III, randomized, double blind, placebo-controlled trial to evaluate the efficacy of already marketed drugs in a new indication. It will be performed at Primary Care doctor visits at several Healthcare Centers in different Spanish Health Areas. A total of 704 non-institutionalized subjects aged 65 years or older will be studied (sample size calculated for a statistical power of 80%, alpha error 0.05, annual incidence of falls 30% and expected reduction of 30% to 20% and expected loss to follow up of 20%). The test drug containing 800 IU of vitamin D and 1000 mg of calcium will be administered daily. The control group will receive a placebo. The subjects will be followed up over two years. The primary variable will be the incidence of spontaneous falls. The secondary variables will include: consequences of the falls (fractures, need for hospitalization), change in calcidiol plasma levels and other analytical determinations (transaminases, PTH, calcium/phosphorous, albumin, creatinine, etc.), change in bone mass by densitometry, change in muscle strength in the dominant hand and change in musculoskeletal strength, risk factors for falls, treatment compliance, adverse effects and socio-demographic data.

**Discussion:**

The following principles have been considered in the development of this Project: the product data are sufficient to ensure that the risks assumed by the study participants are acceptable, the study objectives will probably provide further knowledge on the problem studied and the available information justifies the performance of the study and its possible risk for the participants.

If calcium and vitamin D supplementation is effective in the prevention of falls and fractures in the elderly population, a recommendation may be issued with the aim of preventing some of the consequences of falls that affect quality of life and the ensuing personal, health and social costs.

**Trial Registration:**

ClinicalTrials.gov:  NCT01452243

Clinical trial authorized by the Spanish Medicines Agency: EudraCT number 2006-001643-63.

## Background

Vitamin D insufficiency is common in older people [1] and is associated with several disorders related to aging such as osteoporosis, which leads to a significantly increased risk of bone fractures [2]. These are associated with physical disabilities, reduced quality of life, increased mortality and increase in healthcare costs [3]. According to the SENECA study [4], carried out in elderly people in 11 European countries, 47% of the elderly people had vitamin D deficiency (< 12 ng/ml). This deficiency was more common in Mediterranean countries than in Northern European countries, probably because in the latter countries there is a greater consumption of fish, fortified foods and vitamin D supplements.

According to several authors [5-10], vitamin D serum level is related to muscular strength, physical functional status and risk of fractures and falls, the annual incidence of which is around 30% in persons over 65 years [11]. It has been demonstrated that in institutionalized women, daily calcium and vitamin D supplementation decreases the number of falls and risk of fracture, increases bone mineral density and improves musculoskeletal function [12-14]. The conclusion in a Cochrane review [15], designed to determine the effect of vitamin D on the prevention of fractures in elderly people with senile or post-menopausal osteoporosis, is that the efficacy of this vitamin or of its analogues in the prevention of fractures remains unknown. A reduction in hip fractures in institutionalized, frail, elderly people was observed with the concomitant administration of calcium supplements, but the effect in community-dwelling, younger, healthy older adults was unknown.

According to the result of another systematic review, there is a balance between positive and neutral results [16]. Its use is controversial as it seems clear that is has a positive effect on sub-clinical vitamin D deficiency, resolving a sub-clinical hypovitaminosis state or osteomalacia, but for people living at home not all studies show beneficial effects on bone mass [17]. In their conclusions, the authors of this review state that, overall the evidence analyzed is of intermediate consistency, sufficient for its individualized use, especially in patients with calcium or vitamin D deficiency. However, in order to be able to issue a generalized recommendation, rigorously designed clinical trials are needed, with a sufficient sample size, which can be reproduced in different clinical populations and geographical areas.

Another systematic review [18], aimed at evaluating the effect of vitamin D on strength, physical performance and falls in elderly people in 13 clinical trials (2496 patients), concluded that although there is insufficient evidence that vitamin D supplementation improves physical function in the elderly, some data suggest a benefit of vitamin D combined with calcium. Large, well-designed studies are needed to confirm these findings.

More recently [19], in a meta-analysis performed with a sample of 1237 patients, it was concluded that vitamin D supplementation appears to reduce the risk of falls by more than 20%, both in institutionalized and ambulatory elderly people, although further studies should be considered on the effects of different types and doses of vitamin D and the role of calcium supplements.

There is now general agreement on the importance of vitamin D deficiency in the pathogenesis of osteoporosis and the loss of muscle strength in elderly subjects. However questions are raised on how supplements should be used for prevention in the future. Studies have been performed in different populations with different baseline vitamin D status and different administration regimens, which makes it difficult to compare results [20]. According to forecasts, the population of over 65 years in developed countries will have doubled by 2050, therefore, this will be the age group with the greatest risk of developing osteoporosis and related fractures [21].

Calcium and vitamin D are the mainstay in the treatment of osteoporosis and it is accepted [22] that for women over 65 years with a bone mineral density of -2.5, supplementation (1200 mg of calcium and 800 U of vitamin D) is justified. These can be taken together with other drugs, depending on the presence of risk factors for fracture, the choice of which should be individualized.

Perhaps healthcare policies need to be adopted on the administration of vitamin D supplements in populations at risk of osteoporosis, such as the elderly population and post-menopausal women in Spain and other Mediterranean countries [20]. Vitamin D supplementation could be an attractive public health intervention measure; although further studies are needed before a generalized recommendation can be issued in this respect.

### Study objectives

The first objective is to determine the efficacy of calcium and vitamin D supplementation at doses of 1200 mg and 800 IU, respectively, to reduce the incidence of falls and fractures in non-institutionalized elderly people.

The second objective is to measure and compare treatment groups (calcium and vitamin D vs placebo) as regards muscle strength and musculoskeletal function, bone mineral density, calcidiol level and treatment safety.

## Methods/design

### Design

Phase III clinical trial to evaluate the efficacy and safety of already marketed drugs in a new indication. Parallel group, randomized, double blind, placebo controlled trial.

### Study location and subjects

Patients will be recruited at primary care doctor visits. The subsequent visits will be at the health Centers of the participating Spanish autonomous communities: Castille-La Mancha, Andalusia, the Balearic Islands and Catalonia.

The target population is non-institutionalized patients of 65 years or over. The inclusion criteria are: aged over 65 years with normal renal function, normal transaminase levels, normal calcium blood levels, not homebound (not immobilized) nor in socio-healthcare institutions. The exclusion criteria are: need for medical treatment with calcium or vitamin D, hypersensitivity to or contraindication for calcium or vitamin D, medical treatment that includes calcium or vitamin D, physical disability that impedes their collaboration, taking thiazide diuretics, oral anticoagulants, hormone replacement therapy, digitalis drugs, anticonvulsants or barbiturates and having any of the following diseases: lithiasis, renal impairment (serum creatinine > 1.4 mg/dl), hypo or hyperthyroidism, Paget's disease, chronic liver disease, tumors, sarcoidosis, impaired intestinal absorption or chronic alcoholism (> 40 g/day).

### Sample size

The sample size was calculated statistically to obtain a power of 80% with an alpha error of 0.05. In order to demonstrate an effect in calcium and vitamin D treated patients, consisting of a 30% to 20% reduction in the incidence of falls (relative risk reduction equivalent to 33%), a sample size of 586 (293 in the treatment group and 293 in the non-treatment group) is needed. Assuming a 20% loss to follow-up, 704 subjects between the two groups need to be selected. The two groups will be of equal size in order to obtain the greatest statistical power.

### Blinding and group assignment

After determining that the patients meet the inclusion criteria and do not meet the exclusion criteria, they will be randomized and assigned to the treatment or the control group. The randomization list will be generated by a computer using pseudo-random numbers. Randomization will be centralized. The investigators will receive the information necessary to give each patient the corresponding treatment in blinded conditions. To protect the blinding, the placebo and the test treatment will look exactly the same. The randomization list will contain a patient identification number and the number of the treatment to which he/she has been assigned. This patient number will be used as patient identification in all trial documentation, including the case report form.

To ensure objectivity in measuring the results, the study will be performed in blinded conditions for the patients, the investigators and the data analysts who will not know the labels of the groups they will compare. The placebo and test treatment will have an identical appearance and will only be identified by a code number. The assignment will be unknown by the patient and investigator.

### Intervention

The pharmacological intervention will be the daily administration of chewable tablets containing 800 IU of vitamin D and 1200 mg of calcium. They will be administered over 2 years during the months of November to April in order to avoid the influence of sunlight. Dose adjustment is not contemplated during the study. Concomitant treatment with thiazide diuretics, digitalis drugs, anticonvulsants or barbiturates will not be permitted.

The control drug will be chewable placebo tablets with the same appearance as the test drug.

### Subject follow-up

The subjects will be followed up for a 2 year period. They will be selected at primary care doctor visits, when they will be asked to participate in the study. Once the patients have given their informed consent they will be given an appointment for the randomization visit and an appointment for the analytical tests and densitometry examination before starting treatment.

At the randomization visit the patient will be assigned to one of the study groups, their medical history will be taken, they will be given a physical examination and the study medicine will be dispensed. The following visits will be at 3, 6, 9, 12, 15, 18, 21 and 24 months. At these visits the efficacy (data on falls, fractures, muscle strength and mobility and musculoskeletal function) and safety (the detection of adverse effects) parameters will be collected, treatment compliance will be evaluated and the medication will be dispensed.

Discontinuation of treatment will be for the following reasons: completion of follow-up period (two consecutive years), death, violation of protocol, adverse event, inter-current illness causing immobilization for more than 30 days and patient abandoning trial or withdrawing informed consent.

### Definitions of the primary variables and measuring methods

The primary variable will be the incidence of spontaneous falls according to the FICSIT (Frailty and Injury: Cooperative Study of Intervention Techniques) definition: "Unintentionally coming to rest on the ground, floor, or other lower level. Coming to rest against furniture or a wall was not counted as a fall." Information on the circumstances of the fall (apparent cause, activity performed, time and place and need of help) will be obtained by an interview.

The following are the secondary variables:

- Consequence of falls (bone fractures at any location, need for healthcare, need for hospitalization, bed-ridden).

- Change in calcidiol [25(OH)D3] plasma levels (determined by RIA at baseline and at 6 and 18 months). Vitamin D deficiency is defined as a calcidiol plasma level lower than 10 ng/ml.

- Levels of transaminases, parathyroid hormone (PTH), calcium, phosphorous and serum creatinine, determined by tests on venous blood samples obtained in a fasted state. Calcium/creatinine index, determined by analysis of second morning urine.

- Change in bone mass (bone density or mineral content) obtained by densitometry (risk of fracture). Osteoporosis will be diagnosed based on a densitometry T-score of less than 2.5 in the vertebral column, according to WHO criteria.

- Change in muscle strength in the dominant hand. Determined by dyanometry (with a mean of 3 attempts to obtain a muscle strength measurement).

- Changes in musculoskeletal function, determined by the timed up and go test (the elderly person gets up from a chair with arms, walks three meters, turns round, walks back and sits down again). Taking more than 20 seconds indicates a high risk for falls.

- Stratification and potentially confounding variables; gender, age and other demographic data (social class, education level, marital status).

- Risk factors for falls: taking pre-disposing medication, health problems, toxic habits, physical exercise, living arrangements, sight or hearing difficulties, functional status (Katz index).

- Treatment compliance. To determine this, the patient will bring the medication containers (empty or partially empty) to each visit. According to their degree of compliance (difference between number of tablets dispensed and number of tablets consumed) the patients will be classified as normal compliers (89-100%), hypercompliers (> 110%) or hypocompliers (< 80%). Compliance will also be evaluated by the Morisky-Green test.

- Serious adverse events or any other adverse event. An adverse event is considered as any untoward medical occurrence in any patient included in the study which does not necessarily have a causal relationship with the treatment. An adverse event can therefore be any unfavorable and unintended sign (including an abnormal laboratory finding), symptom, or disease temporally associated with the use of a medicinal product. Known adverse events of the calcium vitamin D combination at the proposed doses are: occasionally slight gastrointestinal alterations and the possibility of stimulating kidney stone formation in patients with impaired renal function.

### Statistical analysis

After performing data cleaning and imputation, exploratory analysis and categorization or transformation of variables the following analyses will be performed:

- Comparison of the variables of interest and the stratification and potentially confounding variables in the treated and non treated groups at the start of the study. We will determine if, despite using randomized assignment, the two groups are homogenous.

- A first raw analysis will be performed to calculate the following parameters and their confidence intervals: ARR (absolute risk reduction), RRR (relative risk reduction) and NNT (number necessary to treat). A stratified analysis will also be performed adjusting for each independent variable. If there are differences between the raw RR and the adjusted RR, the homogeneity between the categories will be determined to establish if the variable is effect modifying or confounding. The incidence of the variables in each group will be described and compared (comparison of proportions by the chi-squared test).

- A survival (fall-free period) analysis will be performed in both study groups (Kaplan-Meier and Log Rank test). Then the effect of treatment on the risk of falls will be determined by constructing a Cox proportional hazards model.

- The effect of treatment on calcium and vitamin D levels will be estimated adjusted for changes in treatment compliance (generalized linear models for repeated measures).

Data will be analyzed on an intention to treat basis including all randomized patients in the efficacy and safety analyses according to their randomized treatment group regardless of the treatment received. A per protocol analysis will also be performed for the primary variables. This will exclude patients who did not meet any of the selection criteria and did not comply with treatment.

For ethical reasons an intermediate analysis will be performed to decide if the study should be discontinued prematurely because of frequent adverse effects occur or an obvious improvement in the test group.

## Discussion

If calcium and vitamin D supplementation is effective in the prevention of falls and fractures in the elderly population, a recommendation may be issued with the aim of preventing some of the consequences of falls that affect quality of life and the ensuing personal, health and social costs.

Using a randomization procedure will reduce selection bias as the investigator does not assign the treatment and any factor affecting participation will be equally divided between the two groups. Confounding is also reduced because randomization tends to share the determining factors that may affect the final result equally among the groups.

However, bias in the collection of information could occur in the study, such as patient evolution, determined by the aging process throughout the two study years, also the regression toward the mean or tendency of the continuous variables to approach the mean value in successive measurements. In our case this is applicable to the number of falls by the elderly people or the analytical test results. Furthermore, the participants, who are aware of the study objectives, could contribute to what is termed "hypothesis guessing" and try to downplay the importance of falls without consequences. There will also be a possible risk of differential loss to follow up between the groups being compared, due to the duration of the study and the placebo effect, which could contribute to patients abandoning the study because they consider the medication they are given is having no effect.

Follow-up visits with controls every 3 months will allow us to balance recommendations on treatment compliance and increase the possibility of detecting adverse effects. This will give us greater control over the development of the study.

As regards problems of external validity, the participants may not represent all eligible subjects if the rate of non responders were high. Furthermore, the study population characteristics (socio-economic level, life-style, etc.) may mean that the results cannot be directly transferred to any other population.

This is a multi-centre study therefore possible sources of error could be: deviation from or misinterpretation of the protocol, obtaining inaccurate or incomplete data and data errors or omissions. To avoid these errors the study personnel will be trained and certified, we will have appropriate documentation, high quality data collection protocols and a data processing system with editing techniques to identify anomalous or erroneous data. To prevent any intentional fraud there will be periodical inspection visits to the participating Centers.

The study protocol has been evaluated and approved by the Investigational Review Board/Independent Ethics Committee of the Albacete Health Area. The following ethical principles will be respected throughout the study: consent to voluntarily participate, guarantee of anonymity of the information provided by the patient, and restriction of the data provided by the interviewee to the proposed investigation. The investigators will ensure that the study will be performed in full compliance with the Declaration of Helsinki in accordance with current legislation (Royal Decree 223/2004) and the New Code of Medical Ethics, approved by the Spanish Medical Association. The study will fully adhere to Good Clinical Practice standards and a civil liability insurance will be taken out to cover any possible harm and injury.

The investigators will be responsible for obtaining written informed consent from the participants once they have adequately explained the objectives, methods, expected benefits and potential risks. The subjects will be informed that they are free to refuse to participate or to withdraw from the study at any time and for any reason.

The following principles have been considered in the development of this Project: the product data are sufficient to ensure that the risks assumed by the study participants are acceptable, the study objectives will probably provide further knowledge on the problem studied and the available information justifies the performance of the study and its possible risk for the participants.

Finally, the study results may only be published in scientific journals or presented at scientific meetings, according to the quality criteria recommended by the CONSORT statement.

## List of abbreviations

FICSIT: Frailty and Injury (Cooperative Study of Intervention Techniques); RIA: Radioimmunoanalysis; I-PTH: Intact parathyroid hormone; ABI: Absolute benefit increase; RBI: Relative benefit increase; NNT: number needed to treat

## Competing interests

The author declares that they have no competing interests.

## Authors' contributions

JL-T is the principal investigator responsible for designing the Project and writing the protocol. JL-T, IPM and FER have participated in calculating the sample size and in the statistical analysis plan. JL-T, IPM, FER, AVF, SMR, JTL, GGF, EVG, VRA and JDA have contributed to the study background, the general design, the definition of the study variables and their adaptation to the computerized clinical record system. All the authors have contributed to the preparation of the project and have read and approved the final manuscript.

## Note

Collaborating researchers (ANVITAD Group): Fernando Andrés Pretel, Rosa Armero Martínez, Esther Balibrea Sancha, Rosario Beltrán Díaz, Clotilde Boix Gras, Carmen Campayo Ortega, Ana Cuenca Abellán, Noé Denia Muñoz, Jesús Díaz Aguado, Francisco Escobar Rabadán, Julia Fernández Sáez, Llanos García García, Mercedes García-Reyes Ramos, Isabel Garzón García, María Dolores González Céspedes, Gerardo Grau Fibla, Pilar Hernández Cano, Llanos Jiménez Atiénzar, Ana López Yeste, Sofía López Cantero, Ángeles Lloret Callejo, Alejandro Manjavacas Garrido, Ángeles Mon Carol, María Isabel Montero Clemente, Julio Montoya Fernández, Susana Morena Rayo, Juana Muñoz Núñez, Beatriz Navarro Bravo, Ignacio Párraga Martínez, José Jorge Pérez Pascual, Susana Pons Vives, Joseba Rabanales Sotos, Alfonso Ramón Bauzá, Marcelino Requena Gallego, Vicente Reyes Adrián, Pilar Sánchez Ortiz, Coral Santos Rodríguez, María José Simarro Herráez, Carmen Somoza Castillo, Juan Manuel Téllez Lapeira, María Ángeles Valero Álvarez, Carmen Vega Fernández, Esther Vilert Garrofa, Alejandro Villena Ferrer

## Pre-publication history

The pre-publication history for this paper can be accessed here:

http://www.biomedcentral.com/1471-2458/11/910/prepub
